# Ultrasonographic abdominal adipose tissue thickness for the prediction of gestational diabetes mellitus: A meta-analysis

**DOI:** 10.17305/bb.2023.9902

**Published:** 2024-08-01

**Authors:** Shuhong Hou, Xiaoqing Xiao, Dongping Chen

**Affiliations:** 1 Department of Ultrasound, Longyan First Hospital Affiliated to Fujian Medical University, Longyan, Fujian Province, China; 2 Department of Internal Medicine, Longyan First Hospital Affiliated to Fujian Medical University, Longyan, Fujian Province, China

**Keywords:** Abdominal obesity, subcutaneous adipose thickness (SAT), visceral adipose thickness (VAT), gestational diabetes mellitus (GDM), meta-analysis

## Abstract

Obesity has been linked to the risk of gestational diabetes mellitus (GDM). The meta-analysis aimed to assess the predictive role of ultrasonographic measurements of the abdominal adipose tissue thickness for GDM in pregnant women. Cohort studies evaluating the association between abdominal subcutaneous and/or visceral adipose thickness (SAT and/or VAT) and subsequent risk of GDM were retrieved from PubMed, Embase, and Web of Science databases. Only studies with SAT/VAT measured before the diagnosis of GDM were included. Random-effect models incorporating the influence of potential heterogeneity were used to pool the results. A total of 13 studies involving 5616 pregnant women were included. Pooled results showed that both a high abdominal SAT (odds ratio [OR] for per 1-cm increment: 1.23, 95% confidence interval [CI]: 1.07–1.41, *P* ═ 0.003, *I^2^* ═ 13%; OR for high vs low category: 3.42, 95% CI: 2.31–5.07, *P* < 0.001, *I^2^* ═ 0%) and VAT (OR for per 1-cm increment: 1.54, 95% CI: 1.16–2.06, *P* ═ 0.003, *I^2^* ═ 63%; OR for high vs low category: 5.73, 95% CI: 3.39–9.77, *P* < 0.001, *I^2^* ═ 31%) at early stages of pregnancy were associated with a higher subsequent risk of GDM. Subgroup analysis based on study design, timing of ultrasound examination, GDM diagnostic criteria, and study quality score showed consistent results. In conclusion, ultrasound-measured abdominal adipose tissue thickness may be useful for predicting the subsequent risk of GDM in pregnant women.

## Introduction

Gestational diabetes mellitus (GDM) is a common comorbidity in pregnant women, with reported prevalence rates ranging from 2% to 38% within the observed population [1–3]. GDM is characterized by the presence of hyperglycemia detected during routine testing conducted between 24 and 28 weeks of pregnancy, failing to meet the diagnostic criteria for diabetes in pregnancy either during or prior to the commencement of pregnancy [4, 5]. From a clinical perspective, GDM significantly elevates the likelihood of maternal hypertension, pregnancy-related hypertensive disorders, eclampsia, and preeclampsia [6, 7]. Furthermore, the incidence of birth trauma, operative deliveries, neonatal respiratory complications, neonatal hypoglycemia, and macrosomia is also heightened by the presence of GDM [7, 8]. Additionally, GDM exerts enduring detrimental effects on both mothers and offspring, raising the risk for type 2 diabetes and various metabolic and cardiovascular abnormalities [1, 9, 10]. Consequently, the timely identification of pregnant women at an increased risk for GDM is of utmost significance.

A growing body of evidence suggests that maternal obesity, particularly abdominal obesity, constitutes a noteworthy risk factor for the development of GDM [11]. Subcutaneous and/or visceral adipose thickness (SAT and/or VAT) in the abdominal region, measured using ultrasound, has recently emerged as a reliable marker of abdominal obesity [12, 13]. This method is preferred due to its non-invasive nature, affordability, and ease of execution, particularly during the first- and second-trimester anomaly screening [14]. However, the potential correlation between ultrasound-measured SAT/VAT in early pregnancy and the subsequent risk of GDM has not been fully determined [15]. Consequently, we conducted a meta-analysis to synthesize the potential predictive effectiveness of ultrasound-measured abdominal adipose thickness for the risk of GDM in pregnant women.

## Materials and methods

Throughout the process of planning, conducting, and reporting the study, the Preferred Reporting Items for Systematic Reviews and Meta-Analyses (PRISMA) statement [16] and Cochrane Handbook [17] were followed.

### Search of databases

We conducted searches in electronic databases, including PubMed, Embase, and Web of Science from their inception to July 28, 2023, to identify studies published up to that date. The search was performed by utilizing terms such as (1) “ultrasound” OR “ultrasonic” OR “ultrasonographic” OR “ultrasonography”; (2) “adipose tissue”; and (3) “gestational diabetes” OR “GDM” OR “gestational” OR “pregnancy” OR “pregnant” AND “diabetes” OR “diabetic” OR “hyperglycemia.” We restricted our inclusion to human studies published in English as full-length articles in peer-reviewed journals. As part of our manual screening process, references from relevant original and review articles were screened for possible relevant studies.

### Inclusion and exclusion criteria of studies

Inclusion criteria were developed in accordance with Population, Interventions, Comparators, Outcomes, and Study (PICOS) recommendations and tailored to the aim of the meta-analysis.

P (Patients): Pregnant women with no previous histories of diabetes who were not diagnosed with GDM at the baseline of the study.

I (Exposure): Early pregnancy measurement of abdominal adipose thickness, including SAT and/or VAT, before the screening for GDM. A high SAT/VAT was considered as exposure. Definitions and thresholds for high SAT/VAT were consistent with those of the original studies.

C (Control): Pregnant women with a low SAT/VAT.

O (Outcomes): Incidence of GDM comparing women with high vs low ultrasound-measured abdominal adipose thickness.

S (Study design): The analysis was restricted to cohort studies which included both prospective and retrospective research.

Exclusion criteria were reviews, editorials, studies not involving pregnant women, studies not assessing ultrasound-measured abdominal adipose thickness, studies not measuring SAT/VAT prior to the diagnosis of GDM, or studies failing to report the outcome of GDM. In cases where patient populations overlapped between studies, the study with the largest sample size was included in the meta-analysis.

### Data extraction and quality evaluation

Literature searches, data collection, and study quality assessments were carried out independently by two authors. In case of discrepancies, the corresponding author was consulted to discuss and reach a consensus. Among the studies included in the analysis, we collected details on study information and design characteristics, the mean age and body mass index (BMI) of the participating pregnant women, the gestational age (GA) for the ultrasonic measuring of abdominal SAT/VAT, methods for analyzing SAT/VAT (as continuous or categorical variables), diagnostic criteria for GDM, the number of women who subsequently developed GDM and the variables adjusted for when evaluating the association between SAT/VAT and the risk of GDM. In terms of quality, the study was scored using the Newcastle–Ottawa Scale (NOS) [18] based on the criteria for participant selection, the comparability of the groups, and the validity of the outcomes. The total score ranges from 1 to 9, with a higher score indicating superior study quality.

### Ethical statement

Ethical approval was not required for this study in accordance with local/national guidelines. Written informed consent to participate in the study was not required in accordance with local/national guidelines.

### Statistical analysis

Odds ratios (ORs) and corresponding 95% confidence intervals (CIs) were used as variables to indicate the association between ultrasound-measured abdominal SAT/VAT and the risk of GDM. ORs per 1-cm increment of SAT/VAT were combined for studies where SAT/VAT was treated as the continuous variable. For studies analyzing SAT/VAT as a categorical variable, ORs comparing the incidence of GDM in women with the highest vs the lowest SAT/VAT categories were pooled. A logarithmical transformation was performed on the risk ratio (RR) measurement and its corresponding standard error (SE) from each study to stabilize and normalize its variance [17]. In order to estimate between-study heterogeneity, the Cochrane *Q* test and the *I^2^* statistic [17] were utilized. An *I^2^* >50% is considered indicative of significant heterogeneity between studies. A random-effects model was applied for pooling the results, as this model accounts for potential heterogeneity [17]. To evaluate how individual studies affected meta-analysis results, sensitivity analyses excluding one dataset at a time [17] were performed. Subgroup analyses were carried out according to study design, GA of ultrasound examination, GDM diagnostic criteria, and study quality scores to determine the influence of the study characteristics on the outcome. Publication bias was assessed using the funnel plot to visually inspect the symmetry, along with Egger’s regression asymmetry test [19]. The statistical analyses were carried out with RevMan (version 5.1; Cochrane Collaboration, Oxford, U.K.) and Stata software (version 12.0; Stata Corporation, College Station, TX, USA).

## Results

### Database search and study retrieval

**Figure 1.**
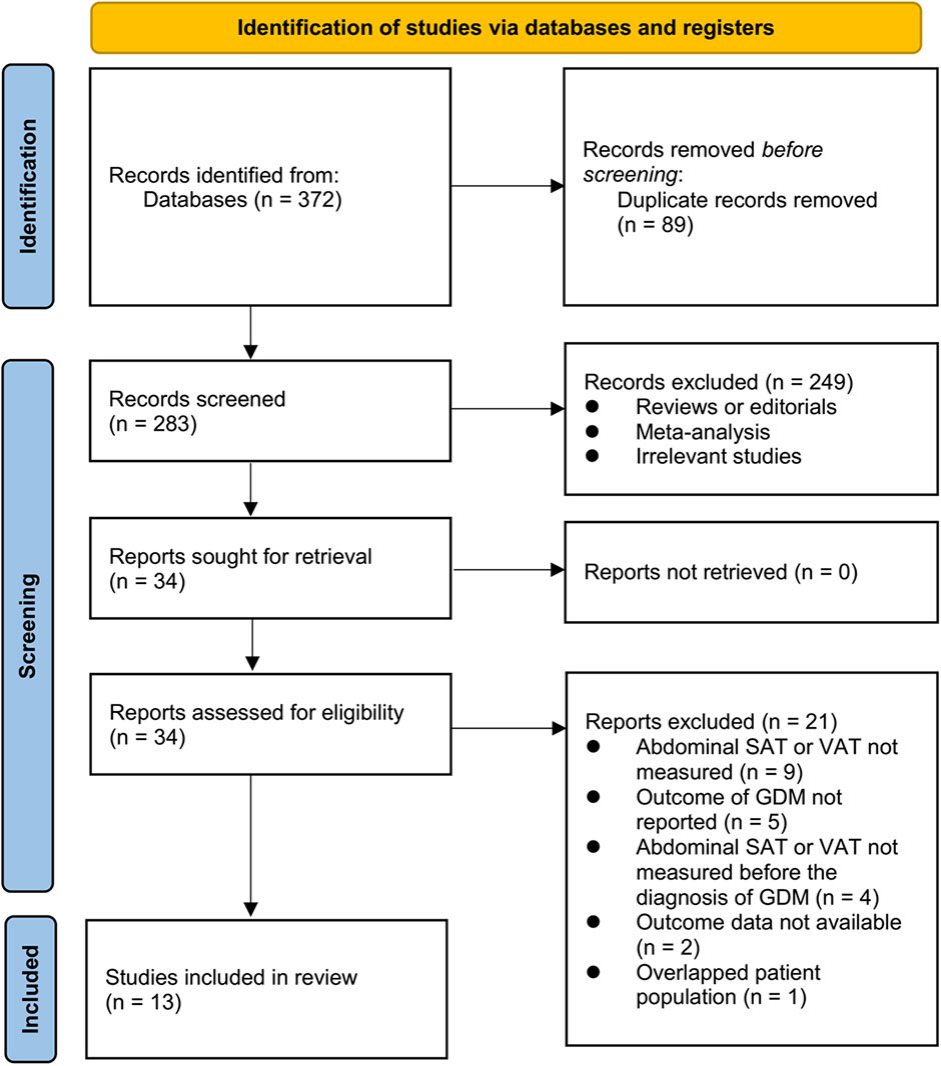
**Flowchart of database search and study inclusion**. SAT: Subcutaneous adipose thickness; VAT: Visceral adipose thickness; GDM: Gestational diabetes mellitus.

Figure 1 shows the process of literature search and study retrieval. Initially, 372 records were obtained from the database and 89 duplicates were subsequently removed. A further 249 studies were excluded based on the title and abstract screening, as they did not fit the meta-analysis’ objectives. Following full-text reviews of 34 studies, 21 were excluded for the reasons listed in Figure 1. Accordingly, 13 studies were obtained for subsequent meta-analysis [20–32].

### Study characteristics

The characteristics of the included studies are summarized in Table 1. Published between 2012 and 2022, these studies were conducted in Australia, Korea, the United States, the United Kingdom, Italy, Brazil, Ireland, Turkey, and India. As for the study design, nine of them were prospective cohort studies [21, 22, 24–30], and four of them were retrospective cohort studies [20, 23, 31, 32]. A total of 5616 pregnant women were included in the meta-analysis. The sample sizes ranged from 83 to 1510. The mean ages of the included patients were 26–33 years, and the mean BMI ranged from 22 to 28 kg/m^2^. All studies conducted ultrasonic measurements for abdominal SAT/VAT within a GA of 9–22 weeks before GDM screening, with 9 of the included studies performing ultrasonic examinations before 14 weeks of GA [21–23, 26–29, 31, 32]. Parameters of abdominal SAT were reported and evaluated as a continuous variable in five studies [20, 22, 25, 27, 29], and as a categorical variable in six studies [21, 23, 24, 30–32]; abdominal SAT were reported and evaluated as a continuous variable in four studies [25–27, 29], and as a categorical variable in seven studies [21, 24, 25, 28, 30–32]. The diagnosis of GDM was based on the International Association of Diabetes and Pregnancy Study Groups (IADPSG) criteria in nine studies [21, 25–32], the American College of Obstetricians and Gynecologists criteria in one study [23], the American Diabetes Association criteria in one study [24], and according to the diagnosis of the medical records in two studies [20, 22]. Variables, including maternal age and BMI, were adjusted in all of the included studies when the association between abdominal SAT/VAT and GDM was reported, while other potential confounding factors such as parity and family history of diabetes were also adjusted to a varying degree among the included studies. The quality scores were between seven and nine stars for the included studies, indicating the good quality of the aforementioned studies (Table 2).

**Table 1:** Characteristics of the included studies

**Study**	**Country**	**Design**	**No. of women included**	**Maternal age (years)**	**Mean BMI (kg/m^2^)**	**GA by ultrasound examination (weeks)**	**Analysis of SAT/VAT**	**Diagnosis of GDM**	**No. of women with GDM**	**Variables adjusted**
Suresh, 2012	Australia	RC	1200	28.8	NR	18∼22	SAT (continuous)	Medical records evidenced	NR	Maternal age and BMI
Kennedy, 2016	Australia	PC	1510	29.3	NR	11∼14	SAT (continuous)	Medical records evidenced	121	Maternal age, smoking, parity, and BMI
De Souza, 2016	Canada	PC	485	32.9	25.1	11∼14	SAT (Q4:Q1) and VAT (Q4:Q1)	IADPSG criteria	45	Maternal age, ethnicity, family history of T2D, and BMI
Yang, 2017	Korea	RC	333	32	22	10∼13	SAT (ROC analysis-derived cutoff)	ACOG criteria	41	Maternal age and BMI
Nassr, 2018	USA	PC	389	30	25.1	18∼20	SAT (ROC analysis-derived cutoff) and VAT (ROC analysis-derived cutoff)	ADA criteria	43	Maternal age, family history of T2D, parity, past bariatric surgery, and BMI
Thaware, 2019	UK	PC	83	32.1	25.6	9∼18	SAT (continuous), VAT (continuous), and SAT (ROC analysis-derived cutoff)	IADPSG criteria	15	Maternal age, parity, education, and BMI
D’Ambrosi, 2020	Italy	PC	295	33.1	22.4	11∼13	SAT (continuous) and VAT (continuous)	IADPSG criteria	57	Maternal age, parity, family history of T2D, and BMI
Rocha, 2020	Brazil	PC	133	26	NR	11∼13	VAT (ROC analysis-derived cutoff)	IADPSG criteria	18	Maternal age and BMI
Alves, 2020	Brazil	PC	518	26.3	24.6	13∼14	VAT (continuous)	IADPSG criteria	87	Maternal age and BMI
Cremona, 2021	Ireland	PC	238	32.6	25.2	10∼16	SAT (ROC analysis-derived cutoff) and VAT (ROC analysis-derived cutoff)	IADPSG criteria	20	Maternal age and BMI
Aydın, 2021	Turkey	PC	142	28.7	26.3	11∼14	SAT (continuous) and VAT (continuous)	IADPSG criteria	19	Maternal age, parity, and BMI
Tunc, 2022	Turkey	RC	100	27.6	28.1	11∼14	SAT (ROC analysis-derived cutoff) and VAT (ROC analysis-derived cutoff)	IADPSG criteria	12	Maternal age, parity, and BMI
Gupta, 2022	India	RC	190	NR	NR	11∼14	SAT (previous study determined) and VAT (previous study determined)	IADPSG criteria	98	Maternal age and BMI

BMI: Body mass index; GDM: Gestational diabetes mellitus; GA: Gestational age; SAT: Subcutaneous adipose thickness; VAT: Visceral adipose thickness; IADPSG: International association of diabetes and pregnancy study groups; PC: Prospective cohort; RC: Retrospective cohort; NR: Not reported; Q4:Q1: The fourth versus the first quartile; ROC: Receiver operating characteristic curve; ACOG: American College of Obstetricians and Gynecologists; ADA: American Diabetes Association; T2D: Type 2 diabetes.

**Table 2:** Quality evaluation of the included studies via the Newcastle–Ottawa scale

**Study**	**Representativeness of the exposed cohort**	**Selection of the non-exposed cohort**	**Ascertainment of exposure**	**Outcome not present at baseline**	**Controlled for age and sex**	**Controlled for other confounding factors**	**Assessment of outcome**	**Sufficient follow-up term**	**Adequacy of follow-up of cohorts**	**Total**
Suresh, 2012	0	1	1	1	1	1	0	1	1	7
Kennedy, 2016	1	1	1	1	1	1	0	1	1	8
De Souza, 2016	1	1	1	1	1	1	1	1	1	9
Yang, 2017	0	1	1	1	1	1	1	1	1	8
Nassr, 2018	1	1	1	1	1	1	1	1	1	9
Thaware, 2019	1	1	1	1	1	1	1	1	1	9
D’Ambrosi, 2020	1	1	1	1	1	1	1	1	1	9
Rocha, 2020	1	1	1	1	1	1	1	1	1	9
Alves, 2020	1	1	1	1	1	1	1	1	1	9
Cremona, 2021	1	1	1	1	1	1	1	1	1	9
Aydın, 2021	1	1	1	1	1	1	1	1	1	9
Tunc, 2022	0	1	1	1	1	1	1	1	1	8
Gupta, 2022	0	1	0	1	1	1	1	1	1	7

### Meta-analysis of the association between abdominal SAT and risk of GDM

**Figure 2.**
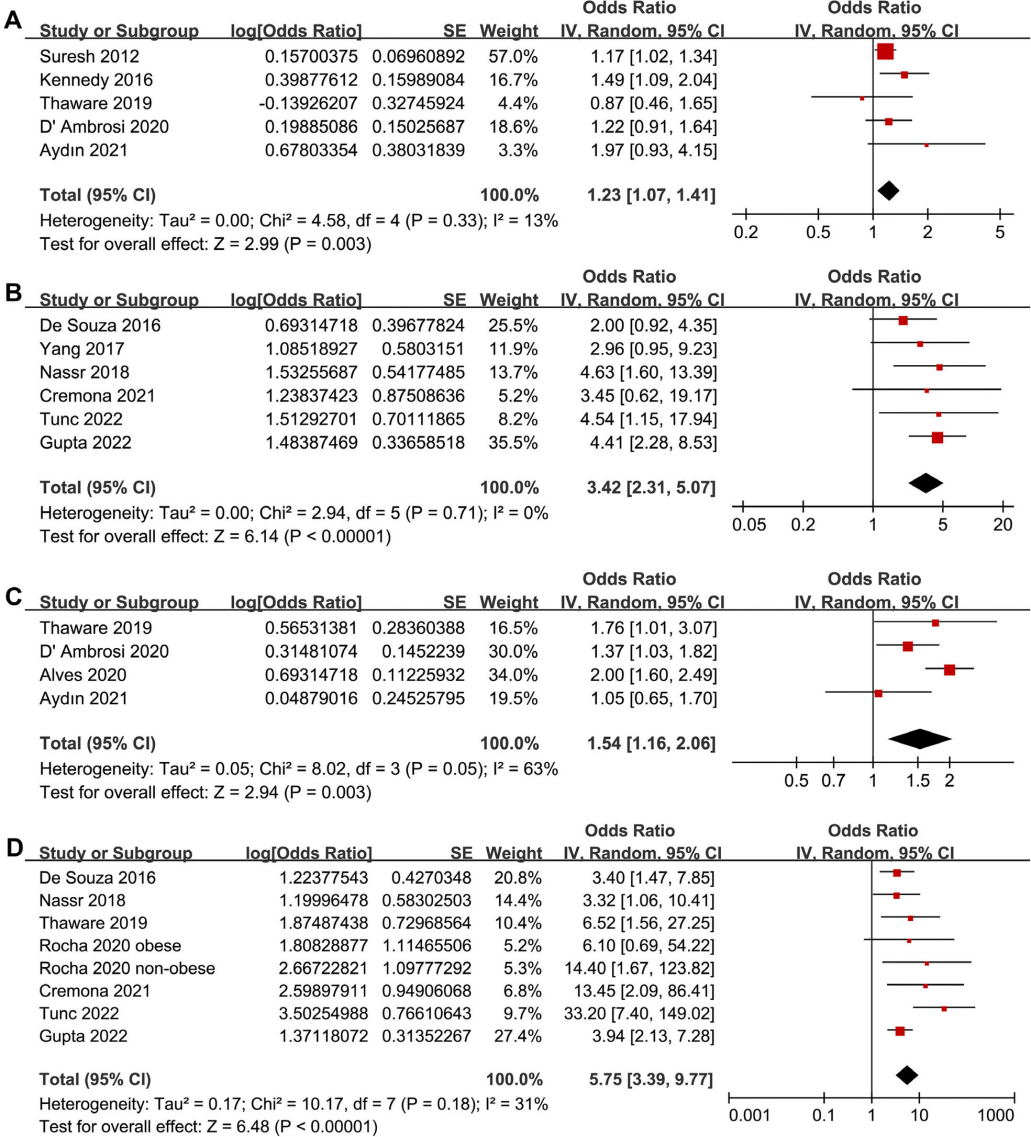
**Forest plots for the meta-analyses regarding the association between ultrasound-measured abdominal SAT/VAT and the subsequent risk of GDM in pregnant women.** (A) SAT analyzed as continuous variable; (B) SAT analyzed as categorical variable; (C) VAT analyzed as continuous variable; (D) VAT analyzed as categorical variable. SAT: Subcutaneous adipose thickness; VAT: Visceral adipose thickness; GDM: Gestational diabetes mellitus; SE: Standard error; CI: Confidence interval.

Pooled results from five studies [20, 22, 25, 27, 29] and six studies [21, 23, 24, 30–32] showed that a high abdominal SAT at early pregnancy was associated with a higher subsequent risk of GDM in pregnant women. This association was found when SAT was analyzed as a continuous variable (OR for per 1-cm increment of SAT: 1.23, 95% CI: 1.07–1.41, *P* ═ 0.003, *I^2^* ═ 13%; Figure 2A) and categorical variable (OR for high vs low category of SAT: 3.42, 95% CI: 2.31–5.07, *P* < 0.001, *I^2^* ═ 0%; Figure 2B). Sensitivity analysis, by excluding one study at a time, showed consistent results (OR for SAT as a continuous variable: 1.18–1.32, all *P* < 0.05; OR for SAT as a categorical variable: 2.98–4.12, all *P* < 0.05). Subgroup analyses, taking into account study design (prospective or retrospective), the timing of SAT measurement (11–14 weeks of GA or others), diagnostic criteria for GDM (IADPSG or others), and study quality score (NOS 7–8 or 9) showed similar results (all *P* for subgroup difference > 0.05; Table 3).

**Table 3:** Subgroup analyses for the association abdominal SAT/VAT and the risk of GDM

**Study characteristics**	**Abdominal SAT as continuous variable**					**Abdominal SAT as categorical variable**				
	**Datasets number**	**OR (95% CI)**	***I* ^2^**	***P* for subgroup effect**	***P* for subgroup difference**	**Datasets number**	**OR (95% CI)**	***I* ^2^**	***P* for subgroup effect**	***P* for subgroup difference**
*Study design*										
PC	4	1.32 (1.05, 1.65)	16%	0.02		3	2.76 (1.53, 4.98)	0%	<0.001	
RC	1	1.17 (1.02, 1.34)	—	0.02	0.37	3	4.07 (2.40, 6.89)	0%	<0.001	0.34
*Timing of measurement*										
11∼14 weeks of GA	3	1.38 (1.12, 1.70)	0%	0.002		3	2.39 (1.31, 4.35)	0%	0.005	
Others	2	1.16 (1.01, 1.32)	0%	0.03	0.16	3	4.48 (2.67, 7.53)	0%	<0.001	0.12
*Diagnosis of GDM*										
IADPSG criteria	3	1.23 (0.88, 1.72)	25%	0.23		2	3.76 (1.73, 8.17)	0%	<0.001	
Others	2	1.26 (1.01, 1.58)	48%	0.04	0.89	4	3.32 (2.10, 5.23)	0%	<0.001	0.78
*NOS*										
7∼8	2	1.26 (1.01, 1.58)	48%	0.04		3	4.07 (2.40, 6.89)	0%	<0.001	
9	3	1.23 (0.88, 1.72)	25%	0.23	0.89	3	2.76 (1.53, 4.98)	0%	<0.001	0.34
**Study characteristics**	**Abdominal VAT as continuous variable**					**Abdominal VAT as categorical variable**				
	**Datasets number**	**OR (95% CI)**	***I* ^2^**	***P* for subgroup effect**	***P* for subgroup difference**	**Datasets number**	**OR (95% CI)**	***I* ^2^**	***P* for subgroup effect**	***P* for subgroup difference**
*Study design*										
PC	4	1.54 (1.16, 2.06)	63%	0.003		6	4.74 (2.76, 8.16)	0%	<0.001	
RC	0	—	—	—	—	2	10.20 (1.28, 81.36)	85%	0.03	0.48
*Timing of measurement*									
11∼14 weeks of GA	3	1.49 (1.04, 2.13)	75%	0.03		5	6.45 (2.93, 14.20)	53%	<0.001	
Others	1	1.76 (1.01, 3.07)	—	0.04	0.62	3	5.34 (2.39, 11.95)	0%	<0.001	0.74
*Diagnosis of GDM*										
IADPSG criteria	4	1.54 (1.16, 2.06)	63%	0.003		7	6.54 (3.55, 12.08)	37%	<0.001	
Others	0	—	—	—	—	1	3.32 (1.06, 10.41)	—	0.04	0.31
*NOS*										
7∼8	0	—	—	—		3	6.15 (2.22, 17.05)	73%	<0.001	
9	4	1.54 (1.16, 2.06)	63%	0.003	—	5	6.04 (2.96, 12.32)	0%	<0.001	0.74

GDM: Gestational diabetes mellitus; GA: Gestational age; VAT: Visceral adipose thickness; IADPSG: International Association of Diabetes and Pregnancy Study Groups; PC: Prospective cohort; RC: Retrospective cohort; NOS: Newcastle–Ottawa scale; OR: Odds ratio; CI: Confidence interval; SAT: Subcutaneous adipose thickness.

### Meta-analysis of the association between abdominal VAT and risk of GDM

Results of meta-analysis incorporating four studies [25–27, 29] revealed that a high abdominal VAT, when assessed as a continuous variable, is associated with an increased risk of GDM (OR for per 1-cm increment of VAT: 1.54, 95% CI: 1.16–2.06, *P* ═ 0.003, *I^2^* ═ 63%; Figure 2C). Sensitivity analysis by excluding one study at a time showed similar results (OR: 1.35–1.70, all *P* < 0.05). Given that the four included studies were prospective in nature, with GDM diagnosed according to the IADPSG criteria, and scored an NOS of 9, subgroup analysis was confined to the timing of VAT measurement. The outcomes remained consistent in studies with VAT measured in 11–14 weeks of GA and those measuring after 14 weeks of GA (Table 3).

Seven studies [21, 24, 25, 28, 30–32] reported the association between abdominal VAT as a categorical variable and subsequent risk of GDM. One study reported two datasets, one for obese and one for non-obese women separately [28]; these datasets were included independently in the analysis. Pooled results showed that a high abdominal VAT as a categorical variable was associated with an increased risk of GDM (OR for high vs low category: 5.73, 95% CI: 3.39–9.77, *P* < 0.001, *I^2^* ═ 31%; Figure 2D). Sensitivity analysis by omitting one dataset at a time showed similar results (OR: 4.37–6.90, all *P* < 0.05). Subgroup analyses according to study design, timing of VAT measurement diagnostic criteria for GDM, and study quality score showed similar results (all *P* for subgroup difference > 0.05; Table 3).

### Publication bias

**Figure 3.**
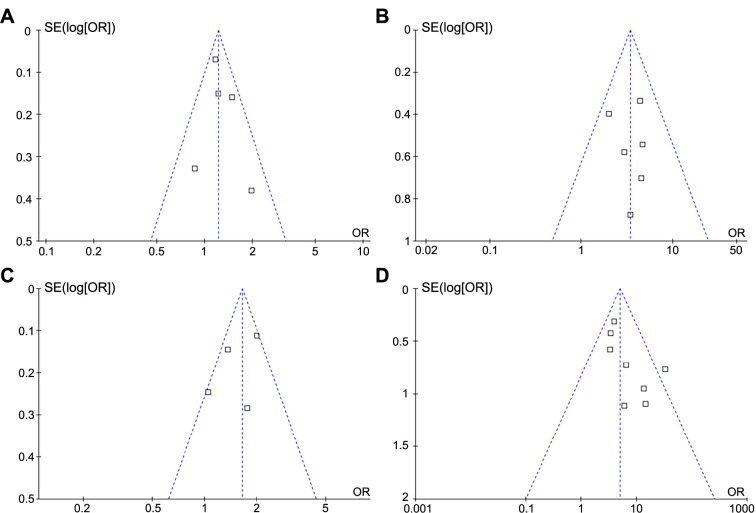
**Funnel plots for the publication bias underlying the meta-analysis regarding the association between ultrasound-measured abdominal SAT/VAT and the subsequent risk of GDM in pregnant women.** (A) SAT analyzed as continuous variable; (B) SAT analyzed as categorical variable; (C) VAT analyzed as continuous variable; (D) VAT analyzed as categorical variable. SAT: Subcutaneous adipose thickness; VAT: Visceral adipose thickness; GDM: Gestational diabetes mellitus; SE: Standard error; OR: Odds ratio.

The funnel plots for the meta-analyses of the association between ultrasound-measured abdominal SAT/VAT in early pregnancy and the subsequent risk of GDM are shown in Figure 3A–3D. The plots are symmetrical based on visual examination, suggesting that publication biases may not be significant. Additionally, Egger’s regression tests could not be conducted due to the limited number of datasets, with only four to eight datasets available for each outcome.

## Discussion

A systematic review and meta-analysis were performed in this study, which incorporated findings from 13 observational studies. The outcomes indicated that pregnant women with elevated abdominal SAT and VAT, as measured through ultrasound during the first or second trimester, were more likely to develop GDM. These findings remained consistent across studies that examined SAT and VAT as both continuous and categorical variables. Additionally, sensitivity analyses, which involved the exclusion of one dataset at a time, further supported the robustness of the results. Furthermore, subgroup analyses indicated that the correlation between abdominal adipose thickness and the likelihood of GDM was not significantly impacted by various study characteristics, including study design, timing of ultrasound examination, diagnostic criteria for GDM, and different study quality scores. In general, the findings of this study provide evidence supporting the utilization of ultrasound-measured SAT and VAT in early pregnancy as a predictive tool for the subsequent risk of GDM in expectant mothers.

To the best of our knowledge, there are limited meta-analyses that have examined the correlation between ultrasound-measured abdominal SAT and VAT with the risk of GDM in pregnant women. A recent meta-analysis conducted in 2021 included seven studies published up until 2020, which investigated the association between visceral adiposity and GDM. The findings of this meta-analysis indicated that the presence of the visceral adiposity phenotype was linked to a significantly higher risk of GDM (OR: 3.25, 95% CI: 2.01–5.26) [11]. However, it is important to note that this meta-analysis utilized a combination of parameters to assess visceral adiposity, including the visceral adiposity index, abdominal SAT, abdominal VAT, and pre-peritoneal fat measurement, making the interpretation of the results difficult [11]. In comparison with the preceding meta-analysis, the present study exhibits numerous methodological advantages. Firstly, a comprehensive exploration of the literature was conducted across three widely utilized electronic databases, yielding 13 contemporary studies that align with the objective of the meta-analysis. Secondly, all the incorporated studies were cohort studies, wherein the measurement of abdominal SAT/VAT occurred prior to GDM screening. Consequently, the findings furnish substantiation that elevated abdominal SAT/VAT during early pregnancy could potentially serve as a predictive indicator for subsequent GDM risk. Thirdly, meta-analyses were conducted independently using ultrasound measurements of SAT and VAT, both as continuous and categorical variables. The consistency of the findings further supports the robustness of the results. Furthermore, all included studies employed multivariate analyses to assess the association between SAT/VAT and GDM, indicating that this association is unlikely to be affected by confounding factors, such as maternal age and BMI. This is particularly significant as both advanced maternal age [33] and high BMI [34] have been linked to an increased risk of GDM.

The conventional parameters that are used to define obesity may possess certain limitations, particularly when applied to pregnant women. While BMI is the commonly utilized measure for obesity, it fails to accurately reflect changes in body composition, particularly during pregnancy. Conversely, the findings of the meta-analysis indicate that a high ultrasound-measured abdominal SAT/VAT continues to be a significant predictor for GDM, even after accounting for maternal BMI. This suggests that visceral adiposity holds additional value in predicting the occurrence of GDM. Waist circumference (WC) is a more accurate indicator of central adiposity and is linked to obesity-related comorbidities. However, it does not differentiate between SAT and VAT, and its reliability is limited during pregnancy due to changes in the abdominal compartment caused by uterine growth. Furthermore, advanced imaging techniques, such as bioelectrical impedance, dual-energy X-ray absorptiometry, and computerized tomography (CT), are considered the gold standard for precisely measuring visceral fat thickness in the general population. Nevertheless, these techniques are costly and cannot be utilized during pregnancy due to alterations in body water redistribution and potential fetus risks of radiation exposure. In comparison to alternative methodologies, ultrasound-measured SAT and VAT exhibit non-invasive attributes, affordability, and simplicity in execution. These measurements have been validated to correlate with parameters obtained through CT and demonstrate favorable inter-observer coefficients of reliability, as well as exceptional reproducibility and repeatability [25]. Consequently, these practical parameters hold promise for predicting the risk of GDM among pregnant women during early pregnancy.

This study has several limitations. First, number of the available studies included in each outcome of the meta-analysis is limited. The results of the meta-analysis should be validated in large-scale prospective studies. Moreover, the optimal cut-off values of ultrasound-measured SAT and VAT for predicting the risk of GDM remain to be determined. In addition, only studies published in English were included in this meta-analysis, potentially leading to publication bias. Besides ultrasound parameters for adiposity, other non-invasive predictors for GDM, such as serum cytokines like placental growth factor have been proposed [35]. Comparative effectiveness between abdominal SAT/VAT vs these serum cytokines for the prediction of GDM should be determined in future studies. Finally, given that this is a meta-analysis of observational studies, a causative relationship between a high abdominal SAT/VAT and the development of GDM could not be derived. It would be interesting to determine if reducing the abdominal adipose thickness through diet and exercise in early pregnancy could reduce the subsequent risk of GDM in pregnant women.

## Conclusion

To sum up, the results of the systematic review and meta-analysis indicate that ultrasound-measured abdominal adipose tissue thickness could serve as a useful predictor for the subsequent risk of GDM in pregnant women, which is independent of the maternal BMI. Although large-scale prospective studies should be performed to validate the findings, these results support the use of ultrasound-measured SAT/VAT in early pregnancy to assess the subsequent risk of GDM in expecting mothers.

## Data Availability

All the data generated during the study are within the manuscript.
